# Book Review

**Published:** 1913-12

**Authors:** 


**Modern Wound Treatment and the Conduct of an Operation.** By Sir George T. Beatson, K.C.B., M.D. Pp. v. 106. Edinburgh: E. & S. Livingstone. 2s. net.—The scope of this little work is fully indicated by its title. It contains a complete and systematic account of aseptic and antiseptic technique. One finds here and there an indication that the author belongs to the conservative school of surgeons. As instances of this tendency, one may mention his non-insistence on the necessity for rubber operating gloves, his preference for antiseptic dressings, even if they are sterilised by high-pressure steam, and the scanty notice of the prominent part played by iodine in modern surgical practice. The recommendation to sterilise silkworm gut and horse-hair by boiling in soda solution must surely be an oversight. But these are small blemishes in a work which, if it contains nothing that a practising surgeon does not carry at his finger-tips, should prove of considerable use to nurses and students commencing clinical work, telling them clearly what to do and why it should be done.

**A Practical Text-Book of the Diseases of Women.** By Arthur H. N. Lewers, M.D., F.R.C.P. Seventh Edition. Pp. xi. 540. London: H. K. Lewis. 1912. 12s. 6d. net. —The fact that this book has successfully reached a seventh edition shows that its merits are recognised. We know that many students appreciate the help in differential diagnosis afforded by the lists of conditions, all presenting some common features, which are to be found scattered through it in their appropriate sections. The present volume is superior to its predecessors in many ways, amongst them in being enriched by numerous micro-photographs of morbid tissues and actual photographs of specimens in the place of the older fashioned drawings. The whole book is brought thoroughly up to date, the description of Wertheim's operation being particularly lucid and the discussion of the indications for operation in cancer of the cervix full and good. The author's arrangement of the subject-matter appears to us to be confusing, and the interpolation in the text of the numerous and detailed case-histories, though these are individually interesting, likely to break into the train of ideas and hinder the grasp of the subject as a whole. Some of the coloured illustrations are good, but in others the colours are not very like those of the actual specimens. Dr. Lewers' style is pleasant and the book easy to read. We predict for the present volume a continuance and increase of the popularity enjoyed by the previous editions.

